# Human liver immunology: from in vitro models to new insights

**DOI:** 10.1038/s41423-025-01312-8

**Published:** 2025-07-02

**Authors:** Milad Rezvani

**Affiliations:** 1 Charité – Universitätsmedizin Berlin, corporate member of Freie Universität Berlin and Humboldt Universität zu Berlin, Department of Pediatric Gastroenterology, Nephrology and Metabolic Medicine, Berlin, Germany; 2 Berlin Institute of Health (BIH) at Charité-Universitätsmedizin Berlin, BIH Center for Regenerative Therapies (BCRT), Berlin, Germany; 3 Berlin Institute of Health – Clinician-Scientist Program, Berlin, Germany; 4 Division of Pediatric Gastroenterology, Hepatology and Nutrition, Cincinnati Children’s Hospital Medical Center, Cincinnati, OH USA

**Keywords:** Liver immunolgy, Organoids, Cell culture, Human in vitro models, Immunology, Translational immunology

## Abstract

The liver hosts a variety of immune cells while creating a tolerogenic environment under homeostatic conditions. However, most chronic liver diseases shift toward inflammation over time. Understanding and intercepting the crosstalk between various immune cells and liver tissue is crucial, as it is often the rate-limiting factor in preclinical drug development. Owing to significant interspecies differences in liver immunology, human models, such as classical cocultures or organogenesis-inspired liver organoids with immune compartments, are becoming essential for advancing the field. Therefore, this review evaluates human-specific models of hepatic-immune crosstalk and assesses a range of models from basic 2D cultures to microphysiological systems (MPSs) and advanced multitissue organoids. It serves as a guide for experimentalists to identify suitable approaches. For example, traditional cocultures offer robustness, reductionist approaches, and modularity but have limited spatial fidelity and cell heterogeneity. In contrast, multitissue organoids inspired by mammalian ontogeny are created from pluripotent stem cells and integrate multiple tissue niche-constituting cells, which include Kupffer-like cells. In conclusion, this review discusses progress in human liver immunology modeling and highlights limitations and numerous untapped opportunities. These include the potential to model in vitro autoimmunity and more complex myeloid inflammatory responses, incorporating contributions from embryonic tissue and bone marrow. Additionally, future in vitro models may include hard-to-culture populations such as neutrophils.

## Background and relevance

The liver is the largest internal organ and a *de facto* immune tissue. In addition to harboring more macrophages than any other tissue, the liver receives blood from a dual vascular inlet, creating a vast endothelial surface area for interaction with circulating immune cells [1]. A diverse range of innate and adaptive immune cells interact with liver tissue within this microenvironment. Kupffer cells (KCs), dendritic cells (DCs), natural killer (NK) cells, T and B cells and many others continuously surveil the liver‒blood interface for antigens, pathogens and cellular debris. The collective hepatic–immune interactions establish an immune-tolerant milieu while clearing pathogens and modulating systemic immunity [2, 3]. In disease, this dynamic and heterogeneous immune environment is disrupted in various ways. Recreating these *in the dish* with sufficient fidelity is a complex task. An increasing number of studies have attempted to model specific hepatic–immune interactions with varying degrees of multicellularity and phenotypic authenticity. This review reviews the literature and evaluates current human-specific models of liver–immune interactions, with a particular focus on untapped future potential.

Although the role of immune cells in shaping hepatobiliary tissue development remains unclear, interactions during adult injury have been extensively studied, e.g., in metabolic-dysfunction-associated liver disease (MASLD) and its progression to steatohepatitis (MASH). However, knowledge gaps remain in, for example, so-called idiopathic inflammatory hepatopathies such as autoimmune hepatitis (AIH), as well as pediatric types hepatitis, e.g. Giant Cell Hepatitis or Idiopathic Neonatal Hepatitis. Most mechanistic insights have traditionally been derived from rodent studies, especially in mice. Unfortunately, critical species-specific differences in human liver immunobiology range from liver architecture and zonation to species-specific drug toxicity, cytokine response and specific immune cell differences. Structurally, liver zonation, including endothelial cell zonation–essential for immune cell adhesion and migration–has been only partially conserved evolutionarily between mice and humans (approximately 60%). This includes differences in the zonated expression of liver-specific lectin that regulates T-cell‒endothelial interactions (CLEC4G) and the recruitment of Th1 and NK cells (CXCL9) [4]. Human-specific endothelial–hepatocyte interactions include the regulation of bile acid metabolism [5]. Another example of poor mouse-to-human correlation is IL-8, which is absent in mice but is critical for human neutrophil recruitment [6, 7]. There is a greater abundance of NK cells in the human liver [8]. Humans and mice share many interspecies core KC-cell signatures, although the typical KC-marker CLEC4F–which is found in many species–is not conserved in humans [9].

Blood and hepatobiliary humanized mouse [10] or human in vitro models [11] (Tables 1 and 2) could help clarify the interspecies liver immunology gap and advance human translational research. Parenchymal and nonparenchymal compartments can, in principle, be humanized in mice [5]. However, such an effort poses logistical challenges related to scalability and throughput. The same limitations apply to human ex vivo-perfused liver [12]–albeit arguably the most authentic experimental environment. As a more readily accessible solution, human in vitro models have gained increasing traction as they increase biological complexity [11]. This is relevant because about 10% all experimental drugs in phase I trials advance to approval, and human toxicity, including hepatic and inflammation-related toxicity, is one of the major roadblocks [as reviewed in 13]. Increasing ethical concerns—especially in Germany—regarding the extensive use of animal models and funding schemes that specifically support human in vitro models enhance the push for a change in preclinical modeling. Regulatory agencies, including the FDA, promote human-relevant in vitro models, such as organ-on-a-chip systems and induced pluripotent stem cells (iPSCs), as alternatives to animal testing in drug development and safety evaluations, as outlined in the FDA Modernization Act 2.0.

**Table 1:** Examples of human-specific hepatic coculture studies

Direct or transwell-based hepato-immune co-cultures				
Ref	Cell source/method	Strength	Main results	Future potential (non-exhaustive examples)
[31]	iPSC-Heps + M1/M0i iPSC-Macs/impermeable transwells	Study cell-contact-independent metabolic effects	M1 Mac-induced insulin resistance	Screens in Hep-or Mac-compartment. Increase Macrophage heterogeneity. Increase transwell exposure time.
[26]	Primary human hepatocytes + autologous hepatic macrophages or THP-1-derived macrophages/direct coculture	Autologous, direct hepatocyte-macrophage interactions	Stability of autologous coculture system for 60 h. Autologous macrophages either exacerbated or mitigated DILI effects.	Patient-specific drug testing, especially with emerging expandable hepatocyte protocols. Dissect human monocyte vs. Kupffer Cell-specific effects
[36]	Intrahepatic cholangiocyte organoids + Primary T_RM_ subset/direct coculture	Modeling autoinflammatory-specific biliary injury	T_RM_-induced FasL & perforin/granzyme-dependent Cholangiocyte apoptosis	Drug-Screening Extending to Other Autoimmune / Autoinflammatory Liver Diseases (e.g. PSC or antibody-mediated diseases, e.g. AIH)
[38]	Cholangiocyte Organoids, LX-2 cells and virally primed MAIT cells/direct coculture	Modeling virally induced autoinflammation in biliary atresia (BA)	MAIT cells promote ductular reaction and fibrogenesis in BA via amphiregulin (AREG)in a TCR-dependent manner	Modulating MAIT cell activity or blocking AREG therapeutically
[39]	Huh-7, HepG3, He3B (EPCAM-high vs -low subpopulation) + primary NK cells/direct coculture	Identifying suppressors of anti-tumor immunity	CEACAM1 limits NK-cell-mediated HCC cell line cytolysis	Enhance efficacy of NK cell-based immunotherapies against liver cancer. CEACAM1 inhibitor development.
[59]	Cholangiocarcinoma (CCA) Organoids + mismatched PBMCs or T cells vs. CCA organoids + immune supernatant/direct coculture	Proof of principle for CCA-immune coculture	Cellular anti-tumor immune responses (increased apoptosis, and elevated release of cytokeratin 19 fragment) Interpatient variability	Autologous coculture. Drug Discovery and Combination Therapies. Model evolution of immune escape.
[41]	HepG2, Huh-7, HepaRG cells overexpressing HDV receptor hNTCP & healthy NK and T cells	Study mechanisms of NK cell activation + cytotoxicity upon HDV hepatocyte infection	IFN-β–TRAIL axis in HDV infection induces innate immune-mediated liver damage, driven by NK cells	Study therapeutic potential (TRAIL inhibitors?). Biomarker discovery. Broader implications: HBV, AIH etc.
**Microphysiological systems**				
[43]	Cholangiocyte Organoids and HLA-matched T cells/direct microfluidic coculture	Study mechanisms of T-cell-mediated liver injury in HCV infection	Organoids pulsed with the HCV nonstructural protein 3 peptide are effectively targeted and killed by patient-derived CD8 + T cells specific to this peptide	Study HCV-related mechanisms in chronic infection. In the future, use expandable primary human adult hepatocytes. Biomarker discovery. Broader implications: HBV, AIH etc.
[44]	HepaRG, LX2 cells. HUVECs, primary or immortalized monocytes (THP1)/Microfluidic two chamber-system, cell-permeable membrane	Study liver sinusoidal, multicellular niche	Invading monocytes restore inflammation-induced Hep-dysfunction, modulate endothelial integrity & promote metabolic recovery	Clinical benchmarking. Use isogenic cell types (e.g. iPSC). Study fibrogenesis.

**Table 2:** Examples of human organogenesis-inspired hepatic-immune models

Organogenesis–inspired Organoids				
Ref	Cell source/method	Strength	Main results	Future potential (non-exhaustive examples)
[20]	Human iPSC-derived hepatocyte-like organoids + macrophage-like & mesenchymal cells /multicellular, self-organising organoid	Multicellular, developmentally guided liver organoid with innate immune and fibrogenic components	Modeled key Metabolic dysfunction-associated steatohepatitis (MASH) features including steatosis, inflammation, and fibrogenesis. Mixed cell culture mediated inflammatory responses to fatty acids and LPS (endotoxin).	Platform for MASH drug discovery (scalable?). Engineer additional immune cell diversity.
[19]	Human iPSC-derived hepatocyte-like organoids harboring KC-like and stellate-like cells + immune stimuli/multicellular, self-organising organoid	Scalable organoid model with inflammatory and metabolic profiling	Identified metabolic and immune-response clusters across MASH patient-derived organoids. Linked genetic risk to inflammatory/metabolic phenotypes.	Personalized organoid-based screening for MASH. Functional validation of genetic variants in metabolic-immune crosstalk. Pooled genotype-phenotype screening for rare diseases.
[21]	Human iPSC/PSC-derived fetal liver-like organoids (FLOs) with a primitive vasculature harboring mesenchyme, hepatobiliary epithelium, and yolk sac–like hematopoietic cells and macrophages/multicellular, self-organising organoid	Models human developmental blood–liver niche with integrated heterogenous hematopoietic and myeloid ontogeny	Integrated heterogenous, yet mostly Yolk sac-like hematopoiesis, into a human liver context. Self-sufficient hematopoietic and hepatic differentiation through crosstalk. Neutrophil-myeloid potential.	Define macrophage–epithelial crosstalk in liver organogenesis. Model early immune disorders. Expand to patient-specific disease modeling and macrophage therapy screening. Study Neutrophil Pathobiology in the Liver.
[22]	Human iPSC-derived hepatic organoids with hepatocyte-like, cholangiocyte-like, stellate-like, and Kupffer-like cells/3D self-organized culture	Multilineage model of liver fibrosis, especially for genetic etiologies, like ciliopathies.	Recapitulated fibrogenic pathophysiology. Antifibrotic PDGFR inhibitor compound reversed fibrogenic response.	Large scale drug testing for fibrosis and inflammation. CRISPR screen Study multicellular fibrotic niches. Add adaptive immune cells for chronic injury models.
[25]	3D liver organoids from human iPSCs-derived hepatocyte-s and M0 + , LX2 stellate-like, and primary cholangiocyte organoids/reconstitution in commercial collagen scaffold (RAFT)	Largely iPSC-based model integrating key parenchymal and nonparenchymal cells for MASLD modeling	Modeled Metabolic dysfunction-associated steatotic liver disease (MASLD) progression from steatosis to inflammation (MASH). Cell phenotypic stabilities.	Platform for therapeutic screening in MASLD in 96 well format. Expand immune diversity. Introduce patient-derived /-specific stellate cells.
[52]	Human iPSC-derived liver organoids integrated with endoderm progenitors and EMPs, giving rise to Kupffer-like cell-harboring liver organoids/codifferentiation and guided progenitor assembly	Modeling EMP hepatic hematopoiesis as in development. Functional Kupffer-like cell incorporation with LPS-responsive transcriptional and secretory profile	Modeled sepsis-induced liver dysfunction. KC-like cell-mediated cytokine responses and hepatocyte injury. Modeled KC-injury recovery. Identified druggable pathways to mitigate inflammation.	Sepsis-on-a-chip liver models. Expand to systemic immune-liver axis. Use for high-throughput drug screening in immune-mediated liver injury.

Human-specific hepatic-immune in vitro assay systems consist of coculture platforms that utilize primary, iPSC, or immortalized human hepatocytes directly with immune cells, such as Kupffer cells, macrophages, and T cells, or behind a solute-permeable but cell-impermeable transwell membrane to study paracrine effects. Another versatile example of coculture with modular biological complexity is liver-on-a-chip microfluidic systems (Table 1). Finally, 3D multicellular organoid systems integrate parenchymal and nonparenchymal components to mimic immune‒liver interactions in vitro (Table 2).

### Coculture versus organogenesis-inspired hepatic immune models

Modeling the complexity of the hepatic-immune niches of the human liver is not trivial. A fenestrated endothelial lining separates circulating immune cells from hepatocytes, allowing for the selective exchange of signals and metabolites. Stellate cells are located on the hepatocellular side of the sinusoidal endothelium in the space of Disse. KCs strategically and dynamically patrol along the sinusoidal side, continuously sample blood-derived antigens and interact with endothelial cells and stellate cell protrusions. Coculture experiments, which are the most straightforward way to mix two cell types, can address reductionist questions with high controllability and reproducibility. However, they do not capture the spatial and architectural principles, mechanical cues, or multicellular feedback of the in vivo microenvironment.

Taking the example of the KC niche, the loss of mandatory niche cues in vitro—and the subsequent loss of KC identity—exemplifies the challenge of reductionist coculture experiments. KCs originate from embryonic precursors, are likely originally derived from the yolk sac, and seed the liver during fetal development, where they become self-maintaining under the influence of the hepatic niche [14, 15]. The long-term identity and function of these cells are shaped by instructive signals from surrounding hepatocytes, liver sinusoidal endothelial cells (LSECs), and hepatic stellate cells, including Notch and BMP signaling. Modeling these spatially and temporally orchestrated signals is not easy. Organogenesis-inspired organoid models derived from human pluripotent stem cells have been shown to exhibit macrophage emergence and maintenance. These have been demonstrated for multitissue organoid models of the intestine [16, 17], brain [18], liver [19–23], yolk sac [24], and other organs, where self-organizing multilineage systems self-organize and mimic aspects of niche architecture. Theoretically, such complex organoids can be fused or cocultured with other organoid systems to model interorgan crosstalk, which emphasizes their potential for future research. Niche-relevant cell types can also be differentiated in parallel and then assembled *a posteriori* in synthetic matrices or engineered microenvironments, such as hydrogels or scaffolds, to recapitulate key instructive signals and spatial organization, e.g., the multicellular response to lipotoxic tissue milieus [25]. Thus, the loss of KC identity in simplified cocultures underscores the difficulty of replicating the complex, spatially and temporally fine-tuned hepatic niche signals *in the dish*, and tissue development-inspired organoid models and engineered microenvironments seek to address this.

Furthermore, modeling direct hepatic–immune interactions is limited by immune incompatibility when modeling crosstalk with circulating immune cells. Allogeneic systems, such as coculturing hepatocytes with mismatched T lymphocytes or NK cells, induce cytotoxicity similar to that in allograft hepatitis. Although organoid-based allograft hepatitis models have significant potential to improve outcomes for transplant patients, they do not reflect homeostasis in healthy patients. The use of autologous material can mitigate this issue. However, obtaining matched autologous liver and immune cells poses significant challenges due to limited access to donor tissues and scalability concerns. Below, we assess the first examples of autologous liver-immune models [26], although they do not address access and scalability. In luminal gastroenterology, primary human intestinal immune organoids were successfully coorganized with autologous tissue-resident memory T (TRM) cells. However, in this paper one of the most critical pieces of information, the freezing media conditions by which tissue-derived T cells are frozen and thawed before their reconstitution, remains undisclosed despite publication in a high-profile paper [27]. In the liver field, this highlights the need for open-access academic research to develop a widely accessible and authentic primary human immune-competent model system.

This review discusses human model systems of hepatic–immune interactions, comparing a variety of coculture models with organogenesis-inspired, multicellular organoid systems. The selection of literature is intentionally nonsystematic and instead aims to provide specific examples from traditional and emerging platforms. Other valuable approaches—such as precision-cut liver slices, decellularized scaffolds, bioprinted models, humanized mouse models or ex vivo-perfused livers—are acknowledged but are mostly covered in other reviews [11]. This review provides conceptual navigation through the literature and assessments for selecting the right model for the right experimental question, understanding that–arguably–no single system can completely recapitulate the human hepatic immune microenvironment yet, although the menu of available liver–immune models is expanding rapidly.

### General considerations: how to choose the right hepatic-immune model

Human hepatic–immune coculture models vary from simple 2D monolayers to complex 3D and microfluidic systems. In 2D, direct mixing or transwell setups allow the study of controllable immune‒parenchymal interactions. However, these methods lack spatial fidelity, but mixing cells at appropriate ratios offers a first approach. Often, finding the right media composition is a topic of discussion, and many researchers end up mixing the hepatic-immune basal media at a 50%-50% ratio while providing key growth factors or cytokines at full concentrations. However, a few iterative steps may be necessary to maintain multiple cell phenotypes in the corresponding media. Paracrine effects can be studied easily if cell-impermeable transwells are used. 3D systems, such as organoids or scaffold-based cultures, can enhance niche signals and potentially improve liver function. However, they may exhibit interexperimental variability as the number of different cell types involved increases. Defined media, ECM, bioreactor-based pooled experimentation, and analytical batch-correcting tools could help improve reproducibility. Dynamic platforms, such as liver-on-a-chip platforms, support flow, zonation, and model immune interactions.

With respect to cell sources, iPSC-derived liver organoids are highly versatile and provide developmental insights, biological complexity, and potential for macrophage integration. Nevertheless, they may exhibit immature phenotypes, although methods exist to improve maturity, such as forcibly expressing transcription factors or adding thyroid hormones to hepatocytes [28, 29]. Adult stem cell-derived organoids offer scalability with more limited cellular diversity. Primary cell-based systems offer physiological relevance but are limited by donor variability and accessibility. Model selection should be guided by the experimental question, balancing complexity, throughput, available resources, and human relevance.

## Liver-immune cell-coculture models

**Fig. 1:**
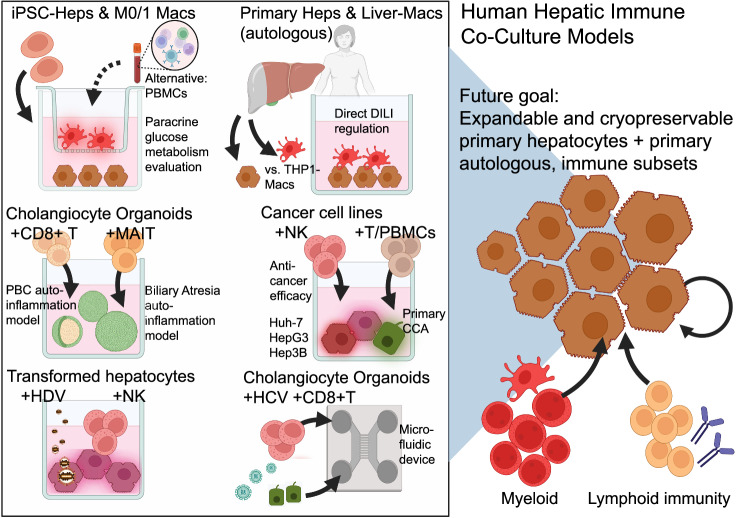
Published examples of human hepatic immune coculture models and future directions (in reference to Table 1). Top left panel: iPSC-Heps cocultured with M0 or M1 macrophages (iPSC-Macs) in a transwell model of contact-independent paracrine signaling, especially for dissecting the inflammatory disruption of hepatocyte glucose metabolism [31]. An alternative setup includes short-term coculture with peripheral blood mononuclear cells (PBMCs) for evaluating polygenic or multifactorial metabolic responses [29]. Top center panel: Compared with THP-1-derived macrophages, primary hepatocytes and liver-resident macrophages (autologously derived from human liver tissue) enable direct modeling of drug-induced liver injury (DILI) [26]. Middle left panels: Cholangiocyte organoids cocultured with CD8^+^ T cells from patients with primary biliary cholangitis (PBC) model antigen-specific T-cell-mediated cholangiocyte injury [36]. Coculture with MAIT cells models biliary atresia-associated fibroinflammatory phenotypes [38]. Middle right panel: Huh-7, HepG3, and He3B cancer cell lines (EPCAM-high vs EPCAM-low subpopulation) were cocultured with primary NK cells, which revealed that CEACAM1 limits NK-cell-mediated HCC cell line cytolysis [39]. Compared with CCA organoids cultured with immune supernatant, CMs cultured with mismatched PBMCs or T cells establish cellular antitumor immune responses and interpatient variability [59]. Bottom center-left panel: Transformed hepatocytes cocultured with NK cells are used for studying hepatitis D virus (HDV) infection and NK cell activation [41]. Bottom right panel: Cholangiocyte organoids (with a hepatocyte hybrid phenotype) were cocultured with CD8^+^ T cells and exposed to HCV peptides using a microfluidic device to simulate adaptive immune-mediated injury [43]. Both figures were created in Biorender.com

Liver immunology research increasingly uses human in vitro models to understand or model the crosstalk between parenchymal and nonparenchymal immune cells. Coculture systems—either direct or transwell systems—offer accessible and modular platforms for studying paracrine and cell contact-dependent interactions (Fig. 1) (Table 1). Unlike organogenesis-inspired liver immune models with ontogenic immune cues, classical cocultures utilize artificial spatial contexts to address questions in a reductionistic manner. Nevertheless, they are relatively easy to conduct and have good reproducibility profiles. In the following, human hepatic–immune coculture models are assessed for studies of metabolism, autoimmunity, cancer and viral hepatitis.

### Paracrine immune triggers of metabolic liver injury in iPSC-based cocultures

Paracrine signals from the endothelium and mesenchyme are known to regulate hepatocyte differentiation, as evidenced by studies comparing direct cellular coculture or contact-independent two-chamber transwells [30]. To understand such contactless paracrine inflammatory effects on human hepatocyte glucose metabolism, the team of Holger Willenbring grew iPSC-derived hepatocytes (iPSC-Heps) with isogenic iPSC-derived macrophages (iPSC-Macs) via a cell-impermeable transwell system [31]. The authors dissected the effect of inflammatory-polarized (M1) vs. undifferentiated (M0) iPSC-Macs on iPSC-Hep metabolism. Through soluble cytokine effects, M1 iPSC-Macs disrupted insulin-dependent glucose and glycogen storage processes in iPSC-Heps, leading to an insulin-resistant cell state, and TNFα and IL-1β were found to be the primary mediators. Unlike many other “fatty liver disease” models, these effects were observed without forced steatosis. As media compositions are key in iPSC cultures, the authors relied on a 50:50 mixture of iPSC-Hep and macrophage basal media. However, the M-CSF concentrations were adjusted to maintain macrophage differentiation.

In this coculture, iPSC-Macs likely had multiple effects on hepatocytes. In addition to impacting glucose metabolism, macrophages likely provide trophic stimuli to hepatocytes [32]. Overall, these findings provide evidence of the impact of macrophage-derived inflammatory feedback on glucose metabolism in the absence of steatotic or lipotoxic milieus. This simple cell-impermeable transwell method shows that it is possible to study contact-independent metabolic effects on cultured hepatocytes and manipulate the cytokines they secrete to identify potential circulating therapeutic targets. However, improving clinical benchmarking could increase its relevance in the clinical setting. While this strategy focuses on a reductionistic binary state of macrophages and excludes other immune cells, it remains versatile, allowing for genetic manipulations or the multiplexing of genetic screens within the hepatocyte or macrophage compartments.

As a related alternative, we demonstrate in a preprint publication the impact of immune cells on metabolic responses in iPSC-Heps. This can also be studied by coculturing patient-derived peripheral blood mononuclear cells (PBMCs) with iPSC-Heps in short-term transwell cocultures. We used this method to dissect multifactorial metabolic liver injuries, including PBMC-derived paracrine effects from other “hits” in hepatocyte injury [29].

In the future, a combination of KC niche-harboring Liver Organoid, along with the flow of circulating immune cells and multiple paracrine factors, would advance the field, making in vitro models more clinically relevant.

### Toward primary liver cell cultures

The apparent goal of many human in vitro models is to recreate the multicellular architecture and functional complexity of human tissues and organs in a controlled, reproducible environment at scale. Georg Damm’s team worked toward this goal. They produce an autologous coculture system of freshly isolated primary human hepatocytes through standard two-step collagenase perfusion, and hepatic macrophages are isolated from collagen-adherent cells found in the supernatant [26]. Such an effort may be feasible only within experimental surgical departments. The hepatocyte-to-macrophage ratio was 4:1 to model the physiologic tissue ratio. Treatment with hepatotoxic drugs caused donor-dependent signs of cell stress and toxicity, with a variable impact on autologous macrophages. Trophic mac-to-hepatocyte effects were also observed. Future research could analyze the roles of classical yolk sac versus bone marrow monocyte-derived macrophages, as their functional differences are increasingly evident [33, 34]. Although donor variability is interesting for patient-specific drug toxicity studies, it hampers reproducibility in other areas. The need for bulk tissue, rather than routine biopsy, makes this co-culture method less accessible to other researchers.

Related to this approach, a recent study demonstrated a seismic breakthrough in the long-term self-renewal of human adult hepatocyte organoids in vitro through the activation of Wnt and STAT3 signaling [35]. Combining this strategy with autologous immune cells is likely to be implemented in the future.

### Modeling T-cell-mediated autoimmunity

Modeling human hepatic autoimmunity in vitro requires the involvement of adaptive immune responses, including autoantibodies and T cells that target liver antigens. Intrahepatic CD8^+^ T-cell subsets display autoreactivity against autologous intrahepatic cholangiocyte organoids from patients with primary biliary cholangitis (PBC) [36]. In patient-autologous cocultures but not control cultures, CD103^+^ tissue-resident memory CD8^+^ T (T_RM_) cells induced cholangiocyte apoptosis, which was identified by the addition of Fas/FasL-dependent and perforin/granzyme blockers. Given the well-established primary cholangiocyte organoid technology [37], another study emphasized the feasibility of modeling biliary-specific autoimmunity: cholangiocyte organoids cocultured with LX-2 stellate-like cells and virally primed Mucosal-associated invariant T (MAIT) cells demonstrated both cholangiocyte proliferation and a fibroinflammatory profile consistent with autoinflammation, fibrosis, and ductular reactions in biliary atresia [38]. Extending such models to hepatocyte-centric autoimmunity, as seen in autoimmune hepatitis, will require expanding human hepatocyte protocols, as recently published [35].

### Modeling anti-cancer-immune interactions

The therapeutic steering of patient immune cells to lyse carcinoma cells is an active field of research. Hepatocellular carcinoma (HCC) cell–immune cocultures may help identify roadblocks to clinical translation. The Huh-7, HepG3 and Hep3B HCC lines were enriched for EPCAM-high and EPCAM-low populations and cocultured with NK cells to assess antitumor activity [39]. CEACAM1 was shown to suppress NK cell-mediated antitumor activity. Consistent with the in vitro results, CEACAM1 blockade treatment in a tumor xenograft mouse model resulted in a favorable decrease in tumor size. A different study combined patient-derived cholangiocarcinoma (CCA) organoids with mismatched peripheral blood mononuclear cells (PBMCs), isolated T cells, or immune cell-conditioned media. A variable antitumor organoid immune response was observed in both systems, especially when T cells were enriched. The supernatants either inhibited or stimulated CCA-organoid growth, underscoring the need for in-depth mechanistic clarification, especially with autologous cocultures. However, establishing matched CCA-immune organoids from clinically stratified cohorts requires significant consortial efforts.

This system enables the assessment of immune-mediated cytotoxicity through direct cell‒cell interactions and soluble factor signaling. This study revealed variable responses across patient-derived organoids, highlighting interpatient heterogeneity and the potential for personalized immunotherapy testing.

### Cancer cell lines in viral hepatitis research

Despite their cancerous nature, transformed hepatocyte lines continue to provide value in researching hepatitis viruses, e.g., hepatitis B [40]. The benefits of these methods are their ease of culture, reproducibility, viral replication and engineering potential. However, aberrant innate immune signaling and insufficient immune niche modeling are limitations. As an incremental progress, HepG2 cells cocultured with peripheral NK cells were infected with hepatitis D virus, which activated NK cells [41]. Using CRISPR/Cas9 gene deletion, this study highlighted the role of TNF-related apoptosis-inducing ligands in the NK cell-mediated killing of infected hepatoma cells. These findings demonstrate the feasibility of immune‒hepatic cocultures for virologic research. Transformed parenchymal and nonparenchymal cell lines, including HepaRG, LX-2, and Mac-differentiated THP-1 cells, may also aggregate to enhance multicellularity. Owing to their cancerous growth, these cell lines can be cultivated in growth factor-free gelatin methacrylate (GelMA) [42]. With a recently published method to expand primary adult human hepatocytes [35], future studies should be benchmarked under such more physiologically relevant cell culture conditions, ideally as immune cocultures.

### Hepatic-immune modeling in microphysiological systems (MPS)

As a step toward primary cell use, human intrahepatic cholangiocyte organoids (ICOs) with hepatobiliary progenitor capacity were cocultured with HLA-matched primary human CD8^+^ T cells via a microfluidic chip embedded in the extracellular matrix [43]. This microfluidic coculture system enabled the modeling of ICO injury when treated with HCV peptides and cotreated with T-cell clones. The strengths of this study include the study of adaptive immune responses to HCV via primary human cells. However, the lack of differentiated hepatocytes and actual HCV infection are limitations.

A team at Jena University Hospital developed a microfluidic multicellular liver model that simulates the human liver sinusoid niche [44]. The researchers utilized biochips of two connected cell culture chambers separated by a cell-permeable membrane. One chamber served as the hepatocyte chamber, which contained immortalized hepatocyte-like cells known as HepaRG cells. The other chamber was designed for nonparenchymal cells (NPCs) and was seeded with a combination of immortalized stellate cells and either primary or immortalized monocytes (THP-1). Monocytes were perfused through the system to assess the inflammatory response to TLR stimulation and its implications for sepsis modeling. Although such a system requires thorough benchmarking of human liver physiology, transferring this model to more physiologically relevant human cells, e.g., expandable primary or well-differentiated iPSC derivatives with and without genetic perturbations, could hold promise. More recently, commercially available alternative solutions have emerged, offering protocols for primary human hepatocyte and NPC MPS cocultures, such as those for predicting drug-induced liver injury [45]. However, more independent academic-based validations are needed.

### Future outlook and potential of hepatic immune cocultures

Human hepatic immune cocultures are an underutilized but promising tool for understanding the complex immunopathologies of liver diseases. Current models offer insights into paracrine and cytotoxic interactions. Nevertheless, gaps remain, particularly in modeling direct cell‒cell communication, autoantibody- and B-cell-mediated autoimmune liver diseases, and the roles of multiple nonparenchymal cell types. The reliance on cancer cell lines continues to limit physiological relevance, underscoring the need for robust, scalable protocols to culture and maintain primary human hepatocytes and immune cells. As the MPSs are increasingly popular, they may offer the necessary flow and compartmental organization to enhance coculture fidelity. Future models should increase fidelity by increasing cell maturity in reductionistic interrogations, e.g., macrophage–hepatocyte crosstalk and multicellularity in complex and chronic pathophysiologies.

## Organogenesis-inspired models of hepatic–immune crosstalk

**Fig. 2:**
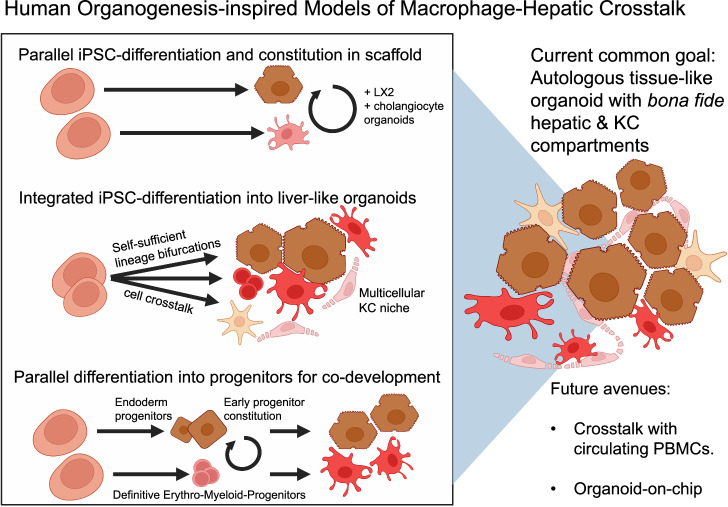
Published human organogenesis-inspired models of macrophage–hepatic crosstalk (in reference to Table 2). Schematic overview of iPSC-based strategies to model human macrophage–hepatic interactions. Approaches include (1) parallel differentiation and aggregation of hepatocyte-like and macrophage-like cells into 3D scaffolds [25], (2) integrated differentiation into liver-like organoids with intrinsic cell‒cell crosstalk [19, 21, 50], and (3) parallel generation of endoderm and erythro‒myeloid progenitors for codevelopment [52]. The common goal is to create autologous tissue-like organoids with bona fide hepatic and Kupffer cell compartments. Future directions include modeling PBMC crosstalk and the development of organoid-on-chip systems

Most current liver organoid systems lack immune cells. The inclusion of bona fide KCs in human liver organoids would be a significant improvement in human liver immunology modeling. Within their native microenvironment, KCs receive instructive niche signals from hepatocytes, stellate and endothelial cells to establish tissue-resident identity [14]. Encouragingly, a first example protocol exists to generate KC-like cells that express appreciable levels of KC-specific markers [46]. However, replication studies, hepatic engraftment studies, and comprehensive benchmarking of human KCs in vivo may be necessary to prove their genuine fidelity, as is also requested for lab-made hepatocytes [47]. The existing benchmarking report of the KC-like cell generation protocol does not clarify the exact contribution of yolk sac- and bone marrow monocyte-derived macrophages. This is relevant since human KCs do not regenerate sufficiently, and circulating monocytes replenish injured KC niches to adopt KC scavenger functions [33]. In theory, existing liver organoids may be combined with iPSC-derived KC-like cells at physiological ratios of hepatocytes to KCs (for example, 10:1-2) to conduct advanced experiments on human-specific hepatic-immune interactions. However, KC phenotype maintenance in vitro is challenging. In a different example, GM-CSF maintained mouse alveolar macrophage identity in ex vivo culture [48]. M-CSF and/or IL-34 (both of which share the same primary receptor) for KCs in vitro are attractive candidates for use in human KC cultures. Another strategy involves incorporating liver sinusoidal endothelial-like and stellate-like cells to provide sufficient KC niche signals. With such a hepatic–immune assembly, experiments could interrogate the hepatic–immune niche, e.g., via knockout screens within the KC or hepatocyte compartment, lineage tracing, or fibro-inflammatory modeling. Such experiments would be particularly attractive for modeling early-onset pediatric diseases because of the developmental characteristics of most iPSC-based organoids [49]. Figure 2 establishes a framework for the following review of the literature.

### Parallel differentiation of hepatocytes and macrophages for 3D reconstitution

Moving toward the goal of a multicellular hepatic-immune niche, Ludovic Vallier and colleagues differentiated hepatocytes and generic M0 macrophages from iPSCs in two separate dishes. After approximately three weeks, the cells were aggregated in a commercially available collagen matrix, and primary cholangiocyte organoids and immortalized LX-2 stellate-like cells were added [25]. Macrophage phenotypes could be maintained in this multicellular mix, arguably owing to BMP-based and Notch ligand-based instructive niche signals from the other cell types, albeit in-depth multicellular niche feedback was not within the scope of this study. To study their combined multicellular response to steatotic or lipotoxic milieus by adding oleic acid (OA) or palmitic acid (PA), respectively, they observed a prompt and generic *TNFa* surge and a late *IL-6* response to PA. Interestingly, the authors also noted human-specific *IL-8* expression. However, human neutrophils capable of responding to this environment are absent, just as endothelial cells are missing. This allows the possibility of establishing fully isogenic, multicellular liver-like tissue in the future, which could even be connected to peripheral blood cells after injury, to model KC niche injury and replacement.

### Integrated multitissue organoid models for hepatic immune inquiries

Efforts to model human tissue development via iPSC/PSC-derived organoids have advanced techniques for interlineage crosstalk. Macrophages and stromal cell progenitor subsets are involved in iPSC-based differentiation of the gut and liver [16, 19, 20]. This strategy could lead to the development of multitissue organoids that are independent of exogenously added cytokines, thereby resolving the challenge of selecting suitable media for various cell types.

Takanori Takebe and team generated an iPSC-derived hepatic liver organoid model (HLO) that includes hepatocyte-, stellate-like-, and Kupffer-like lineages, enabling the recapitulation of steatohepatitis-like pathology, including a fibroinflammatory response [20]. Similar organoids have been used for massive genotype‒phenotype studies relevant to MASLD and drug-induced cholestasis studies [19, 50]. These KC-like cell-harboring organoids release inflammatory cytokines (e.g., IL-6, TNFα, and IL-8). Remarkably, a preclinical drug candidate that enhances intracellular NAD+ de novo synthesis recapitulates the therapeutic metabolic effects observed in an in vivo mouse model of MASLD/MASH in HLOs. Interestingly, the drug was primarily effective in iPSC lines harboring a single-nucleotide polymorphism in the glucokinase regulatory protein (*GCKR*) gene [51]. These findings demonstrate the translational potential of precision medicine, such as for MASH, in multifactorial inflammatory diseases.

To better understand the cellular contribution to macrophage development and overall inflammation, we continued this extensive work by coinducing hepatic and hematopoietic development in human fetal liver-like organoids (FLOs, see preprint [21]). Hepatocyte differentiation requires only early inductive exogenous signals, indicating that intrinsic hepatocyte-differentiating organoid crosstalk occurs later during organoid development. Spontaneous macrophage differentiation likely originates from heterogeneous, primarily yolk sac-like hematopoietic cell differentiation influenced by intrinsic, mesenchyme-derived M-CSF. The addition of additional recombinant cytokines can expand and promote the acquisition of mature macrophage phenotypes. Generally, FLO-treated macrophages exhibit anti-inflammatory polarization. A subset of FLO-derived macrophages expressed KC markers, including *VSIG4, MARCO*, and *FCGR3A*. Despite strong myeloid bias, FLOs retain fetal B and T-cell potential, opening the door for future autologous hepatic adaptive autoimmunity modeling. As a case study to codevelop neutrophils, which are notoriously challenging to study in vitro because of their high turnover, we modeled a neutrophil-driven lipotoxic injury response, including human-specific IL-8 secretion. Thus, liver models involving complex myeloid participation are being developed.

### Aggregating hepatocyte and kupffer cell progenitors

Alternatively, a team surrounding Hideki Taniguchi generated iPSC-derived liver organoids from iPSC-derived erythromyeloid progenitors (EMPs), the origin of tissue-resident macrophages, and iPSC-derived endoderm [52]. This is an attractive solution because, during embryonic ontogeny, EMPs derived from hemogenic endothelial cells in the yolk sac engraft in the liver and give rise to Kupffer cells. There was no multicellular KC niche, and accordingly, M-CSF had to be added. The authors could model hepatic-immune dysfunction during sepsis by adding lipopolysaccharide (LPS) and interferon-gamma (IFN-γ). Interestingly, the cessation of sepsis-like injury led to a regenerative recovery response within both KC-like and hepatocyte-like cell compartments, raising the question of what would have occurred in the presence of circulating monocytes–possibly replacing KCs–or whether this is a feature of the fetal or newborn human liver, which may respond more plastically. This is relevant because human KCs are considered self-maintaining and long-lived, but they have a limited regenerative response. This finding highlights the therapeutic potential of the transplantation of liver organoid-derived KCs, which is related to ongoing autologous macrophage trials by Stuart Forbes [53]–which harnesses M-CSF ex vivo-treated peripheral blood-derived macrophages. Interestingly, treatment with a TLR4 inhibitor mitigated sepsis-like injury in the organoids described above, especially when it was started early, suggesting another therapeutic approach.

### Outlook and potential of organogenesis-inspired hepatic immune cultures

Organogenesis-inspired liver models are evolving as tools to recapitulate the multicellular complexity of the hepatic immune niche. The field is moving beyond hepatocyte-centric systems by integrating iPSC-derived macrophages–some with KC-like features–into 3D organoids. Thus far, reports have enabled the modeling of early metabolic-inflammatory triggers such as lipotoxicity or pathogen-associated molecular pattern exposure and the interrogation of the first proof-of-principle examples or therapeutic responses in human genetic contexts.

Despite this progress, most of these human models capture only basic aspects of hepatic inflammation. One example is the inability to mimic the dynamic interplay between tissue-resident KCs and monocyte-derived liver macrophages following injury. Furthermore, lymphoid cell lineages are absent (but have been included in multiple coculture experiments, see above). Modeling adaptive immunity—including autoreactive T and B cells and plasma cells relevant to autoimmune hepatitis or primary sclerosing cholangitis—is unexplored mainly in these organogenesis-inspired models owing to persistent challenges in lymphoid lineage induction from iPSCs/PSCs. Similarly, human NK cells, γδ T cells, and mucosal-associated invariant T (MAIT) cells have yet to be integrated. Neutrophil biology is also insufficiently understood despite its central role in acute liver injury.

Developmental protocols show promise for future potential but remain technically challenging, variable, and difficult to scale. Critical issues include inconsistent immune maturation, nonphysiological cell ratios, and limited organoid longevity, although the modeling of pediatric diseases, especially cholestatic autoinflammation, has enormous untapped potential. Notably, these types of multicellular liver organoids are still actively being revised, such as their modeling with placenta-derived factors, which could be harnessed to improve hepatoblast expansion in vitro and correct the current cell ratios of existing models [54].

Generally, next-generation organogenesis-inspired liver organoids should include both yolk sacs and be combined with readily accessible peripheral blood or bone marrow-derived immune cells, involve spatially resolved coculture setups, and integrate dynamic peripheral immune cell influx.

## Concrete possible applications and limitations

Immune-competent models of human liver pathobiology could help overcome major clinical challenges, e.g., diseases with multifactorial, complex pathophysiology. Concrete examples are models for metabolic dysfunction-associated steatohepatitis (MASH), autoimmune hepatitis (AIH), and alcoholic liver disease (ALD). As a specific example, the use of human liver models for understanding ALD has been widely underutilized, especially since this field continues to rely heavily on rodent models. Despite hepatocellular immaturity, organogenesis-inspired models [21, 49, 50] could already be used to model innate cytokine-dependent pathways (e.g., IL1-Beta), fibrogenesis, steatosis, macrophage polarization switching and possibly neutrophil crosstalk [55]. However, adequate benchmarking, ideally to human ALD tissue, will be required to improve pathophysiological relevance, e.g., using a combination of functional and single-cell genomic data integration. Molecular benchmarking to precision-cut liver sections is another idea [11]. Additionally, comparisons of human in vitro ALD-induced inflammation models with common mouse models of immunity would be valuable to the field. In particular, an interspecies assessment of neutrophil recruitment in ALD [6], which differs in mice, could help to review past literature in a new light. For MASLD and its progression to MASH, there has been increasing use of human in vitro models. However, the role of neutrophil pathobiology, the impact of the gut‒liver axis, systemic cues, and zonation modeling could present critical new avenues of investigation. A new protocol to assemble different zonated human liver organoids into a multizonal microtissue organoid holds significant potential for MASLD research [56]. This is important because, in MASLD, substrate excess and other compounded injuries may disrupt normal zonation, traditionally injuring and inflaming the pericentral region. Additionally, pediatric populations may also exhibit periportal injury patterns. As mentioned above, while myeloid immunity modeling of liver diseases is becoming more realistic, adaptive liver immunity modeling is still poorly established. Here, cellular autoinflammation is a more realistic model than autoimmunity, as it requires antigen-specific adaptive responses central to AIH. Future progress will depend on integrating functional lymphocytes and modeling antibody-mediated autoimmunity. In the future, TCR sequencing of organoid-derived T cells (as we are developing [21]) could reveal clonal expansions and autoreactive signatures, helping to validate and refine models of adaptive liver autoimmunity. It enables tracking of antigen-specific responses and could guide testing of immunotherapies in autologous settings. As inspiration for the future of AIH modeling, autologous gut organoids from endoscopic biopsies preserve the epithelium, native mesenchyme, and tissue-resident immune cells as a unit, enabling the modeling of celiac disease, an autoimmune disease of the small intestine, to identify functional pathways without cellular reconstitution, as in current cocultures [57].

## Conclusion: future directions and perspectives

This review examines recent human model systems that study liver–immune interactions and highlights the need for human-specific platforms, given the species-specific differences in liver immunobiology between mice and humans. It explores a spectrum of models, from reductionistic 2D cocultures and transwell assays to advanced 3D organoids and microphysiological systems (MPSs), including liver-on-a-chip devices. This review surveys traditional coculture models that offer modularity but limited spatial fidelity and contrasts them with organogenesis-inspired multicellular liver organoids derived from iPSCs that aim to recapitulate tissue development and niches and include, thus far, macrophage lineages such as KC-like cells. Examples of both types of models span from metabolic, especially MASLD, disease modeling to viral hepatitis, autoimmunity, and cancer immunology. However, a broader range of indications has been used with traditional cocultures. This may be due to the resources required for organogenesis-like, multicellular organoid development, variability in results, and difficulty in benchmarking against native tissue, among other reasons.

Experimentalists in liver immunology are advised to understand the strengths and weaknesses of available human models. In the future, a variety of human-specific CRISPR screens related to human inflammatory hepatopathies can be developed, as well as fundamental questions, such as how immune cells shape the development of the liver parenchyma, which seem obvious but remain largely unknown. The accurate modeling of autoimmune or autoinflammatory liver diseases, e.g., AIH, PSC and BA, remains a major need, and a combination of the culture methods above could be exploited. Expanding adult hepatocyte protocols is another critical need. A significant need is the standardization and common benchmarking of increasingly complex organoids, and a unified nomenclature is an excellent example despite the disagreements and competition in the field [58]. The extensive blood‒liver humanization of mouse models is another untapped potential, albeit mouse organismal contributions may remain. Beyond cell culture models, the use of ex vivo-perfused human livers may become the gold standard, although access to this technology seems to be the main hurdle [12]. Finally, appropriately benchmarked data on hepatic–immune crosstalk could eventually be integrated into fully in silico -simulated multicellular models.
